# Improved Method for DNA Extraction and Purification from *Tetrahymena pyriformis*

**DOI:** 10.3390/mps2020040

**Published:** 2019-05-15

**Authors:** Ezzouhra El Maaiden, Youssef El Kharrassi, Abdel Khalid Essamadi, Khadija Moustaid, Boubker Nasser

**Affiliations:** 1 Laboratory of Biochemistry and Neurosciences, Department of Biology, University Hassan I, BP 577, 26000 Settat, Morocco; zohra.elmaaiden@gmail.com (E.E.M.); elkharrassiyoussef@gmail.com (Y.E.K.); essamadi2002@yahoo.fr (A.K.E.); 2 Laboratory of Applied Chemistry and Environment, Department of Chemistry, University Hassan I, BP 577, 26000 Settat, Morocco; moustaid_khadija@yahoo.fr

**Keywords:** *Tetrahymena pyriformis*, DNA extraction, SDS 20%/Triton X-100, Chelex^®^ 100 matrix, APD complex, SDS-chloroform

## Abstract

*Tetrahymena pyriformis* (protozoa) is intensely investigated as a model organism, offering numerous advantages in comprehensive and multidisciplinary studies using morphologic or molecular methods. Since DNA extraction is a vital step of any molecular experiment, here a new mixed surfactant (Sodium dodecyl sulfate (SDS) 20%/Triton X-100) was adopted for effective DNA extraction from *Tetrahymena pyriformis* under an easy, fast protocol. The efficiency of this technique was then compared with three widely-used alternative techniques, namely the Chelex 100 matrix, Ammonium pyrrolidine dithiocarbamate (APD) complex and SDS–chloroform methods. DNA extraction was analyzed by pulsed-field gel electrophoresis, spectral measurement, fluorometry (Qubit), restriction enzyme digestion, and polymerase chain reaction. Data analysis revealed that the quantity and quality of the recovered DNA varied depending on the applied DNA extraction method. The new method (SDS 20%/Triton X-100) was the most efficient for extracting DNA from *Tetrahymena pyriformis* with high integrity and purity, affordable cost, less time, and suitability for molecular applications.

## 1. Introduction

The ciliate *Tetrahymena pyriformis* is a cell model for cellular and molecular biology [1,2]. Like other ciliates, this species has separate germline and soma functions that are embodied by distinct nuclei within a single cell. The germline-like micronucleus (MIC) has its genome held in reserve for sexual reproduction. The soma-like macronucleus (MAC), which possesses a genome processed from that of the MIC, is the center of gene expression, and does not directly contribute DNA to sexual progeny. High-quality isolation and purification of DNA is the first and most undervalued step in molecular biology methods, and depends on the high purity and concentration of extracted DNA [3]. On the other hand, the process should be safe, simple, quick, and low-cost. Finally, the DNA extraction technique chosen should be able to deliver pure DNA samples ready to be used in downstream molecular applications [4]. The basic steps of DNA isolation are (1) the disruption of the cellular structure to create a lysate, (2) the separation of the soluble DNA from cell debris and other insoluble material, and (3) the purification of the DNA of interest from the soluble proteins and other nucleic acids [5]. The elimination of materials by surfactants is one of the most favourable methods of purification, due to their excellent binding ability with a variety of biomolecules and ions [6]. This paper describes a simplified and quick method of DNA extraction from *Tetrahymena pyriformis* using a new mixed surfactant (sodium dodecyl sulfate (SDS) 20%/Triton X-100). The efficiency of this technique was compared with three widely-used alternative techniques, namely the Chelex 100 matrix, Ammonium pyrrolidine dithiocarbamate (APD) complex and SDS–chloroform methods.

## 2. Experimental Design

**Figure 1:**
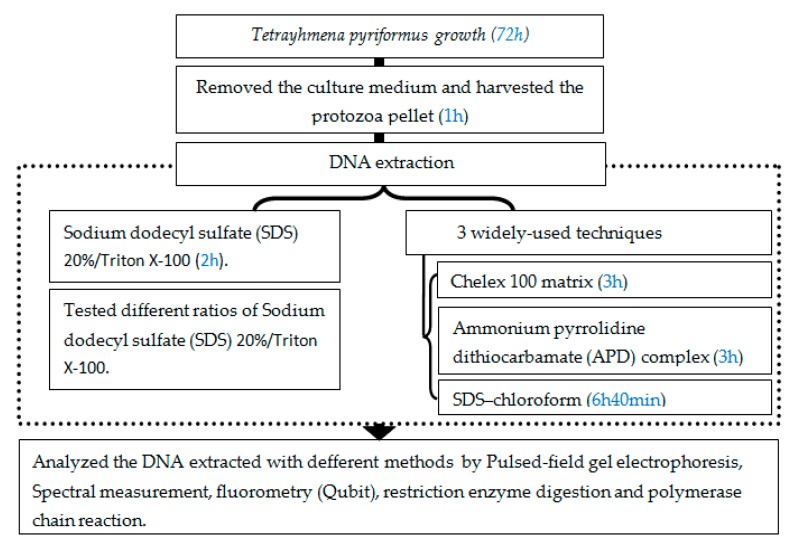
Experimental design for the extraction of DNA with sodium dodecyl sulfate (SDS)/Triton X-100, and the comparison of its quality and quantity with other widely-used methods.

Our method consists of the following steps, as shown in Figure 1. The first step was the culture of our strain of *Tetrahymena pyriformis (GL, L 1630/1)* axenically in the standard medium PPYE (proteose peptone-yeast extract). The culture medium PPYE was removed and the protozoa pellet was submitted to DNA extraction with the SDS/Triton X-100 method. We needed to determine the ratio of SDS/Triton X-100 that provided a high quality and quantity of DNA, so different ratios of SDS/Triton X-100 were tested (0/100, 20/80, 40/60, 60/40, 80/20, and 100/0); the ratio that provided the highest quality and yield of DNA was compared with three widely-used techniques (Chelex 100 matrix APD complex and SDS–chloroform).

### 2.1. Materials

The following materials were used in the experiment:

- SDS 20% solution (Thermo Fisher; Waltham, MA, United States; Cat.no.:AM9820);
- Chelex 100 matrix (BIO-RAD; Hercules, CA, United States; Cat.no.:1421253);
- Tris, 1 M, pH 8.0 (Thermo Fisher; Waltham, MA, United States; Cat. no.: AM9855G);
- Ethylene diamine tetra acetic acid (EDTA, Thermo Fisher Scientific; Waltham, MA, United States; Cat. no.: 17892);
- Ethanol 100% (VWR chemicals, VWR, Radnor, PA, United States; Cat. no.: 64-17-5);
- Proteinase K (Thermo Fisher Scientific; Waltham, MA, United States; Cat. No.: EO0491);
- Agarose molecular grade (Roche, Burgess Hill, United Kingdom; Cat. no.: 11388983001);
- Tris- Ethylenediaminetetraacetic acid (EDTA), (TE, St. Louis, MI, United States; Cat. No.: 99302);
- Restriction endonuclease HindIII (Thermo Scientific, Waltham, MA, United States; Cat.no.:ER0501);
- GeneRuler 1 kb DNA Ladder (Thermo Scientific, Roskilde, Denmark; Cat.no.:SM0311);
- Primers MT (see Table 1);
- Ethidium Bromide (Thermo Fisher; Wilmington, DE, United States; Cat.no.: 10342020);
- Deoxynucleotide (dTNP) mixture (25 mM) (Takara bio; Shiga, Japan; Cat.no.: 4030);
- Taq Polymerase; (Thermo Fisher; Naerum, Denmark; Cat.no.: 10342020);
- Manganese (II) chloride (Thermo Scientific; Karlsruhe, Allemagne, Cat.no.: 7773-01-5);

### 2.2. Equipment

The following equipment was used in the experiment:

- Microcentrifuge tubes 1.5 mL (Eppendorf, Hamburg, Germany; Cat. no.: T9661-500EA);
- Pipette-tips, 10 µL, 200 µL, 1 mL (Axygen, Union City, CA, United States; Cat.no.: 14-222-690, 14-222-812 and14-222-737);
- Shaking heat block (Thermomixer, Eppendorf; Hercules, CA, United States, Cat.no.: 363000233);
- Water bath (JULABO TW9; JULABO, Seelbach, Germany; CAT.no.: 9550323);
- Vortex (Genie 2; Scientific Industries, Bohemia, NY, United States; Cat.no.: SI-T236);
- Laboratory scales (Sartorius, Göttingen, Germany; Cat.no.: ENTRIS623i-1S);
- pH meter (HANNA Edge HI2020-02; HANNA, Woonsocket, RI, United States; Cat.no.: HI2020-20);
- Homogenizer (Bullet Blender; Next Advance; Bohemia, NY United States; Cat.no.: 344-0014);
- Autoclave (HIRAYAMA, Saitama, Japan; Cat.no.: 344-0014);
- Microcentrifuge (Eppendorf; Cat. No.: 5424R);
−20 °C freezer (Thermo scientific™; Goteborg, Sweden; Cat.no.: TSX2320FD);
- 4 °C refrigerator (Nor-Lake™, Goteborg, Sweden; Cat.no.: 22-650-528);
- MilliQ water purification system (Merck Millipore, Burlington, MA, United States; Cat.no.: ZIQ7003T0);
- Gel electrophoresis system (Bio-Rad, Hercules, CA, United States; Cat.no.: 150-0739);
- NanoDrop One Microvolume UV-Vis Spectrophotometers (Thermo Fisher Scientific; Wilmington, DE, United States; Cat.no.: 13-400-519);
- BD Accuri C6 Plus Flow Cytometer (BD Biosciences; Hercules, CA, United States; Cat.no.: 170-3612);
- Qubit fluorometer (Thermo Fisher; Hercules, CA, United States; Cat. no.: Q33226);
- Molecular Imager Gel Doc XR System (BIO-RAD; Hercules, CA, United States; Cat.no.: 1708195EDU);
- Chef-DR II Gel Electrophoresis System (BIO-RAD; Hercules, CA, United States; Cat.no.: 170-3612);
- Polymerase chain reaction (PCR) tubes (Axygen; Hercules, CA, United States; Cat. no.: 14-222-262);
- Rotaphor (Biometra; Gottingen, Germany; Cat.no.: 846-021-101);
- Thermocycler (Super Cycler SC300G, Kyratec, Mansfield, Australia; Cat.no.: SC-200);

## 3. Procedure

### 3.1. Strains and Culture Conditions

**Figure 2:**
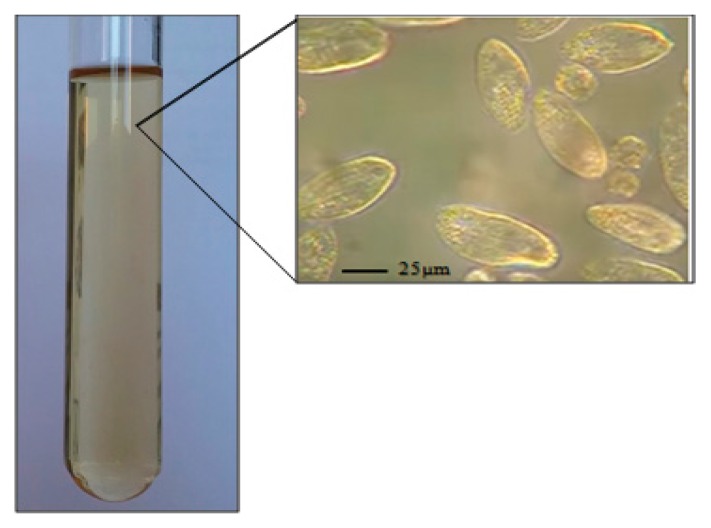
Microscopic image of *Tetrahymena pyriformis (GL, L 1630/1)* in proteose peptone-yeast extract medium (×100).

- *Tetrahymena pyriformis* (strain *GL, L 1630/1*, from The Culture Collection of Algae and Protozoa, The Botany School, Downing Street, Cambridge) was cultured axenically in 5 mL of the standard medium (PPYE), containing proteose peptone (1.5%, w/v) and yeast extract (0.25%, w/v) (Figure 2).
- The culture was then inoculated with 1% (*v*/*v*) *Tetrahymena pyriformis (GL, L 1630/1)* preculture in the same medium and incubated at 28 °C without shaking for 72 h [7].
- At the end of the incubation time, the culture (6 × 10^6^ cells/mL) was then harvested at the exponential phase by centrifugation at 5000 rpm for 5 min at 4 °C.
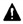
**CRITICAL STEP** To remove the medium completely, decant the medium from the centrifuge tube after centrifugation, invert the centrifuge tube on a paper towel to remove any residual liquid, then tap the tube gently on the paper towel to remove any liquid stuck to the sides of the tube.
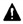
**CRITICAL STEP** To get more culture for DNA extraction, repeat the above process by adding more culture to the same centrifuge tube.
- After removing the medium, wash the protozoa cells with 500 μL ice cold Tris–EDTA (TE) solution (Tris–EDTA: 100 mM Tris, 10 mM EDTA, pH 8.0), resuspend the protozoa pellet by vortexing or slow rounds of pipetting, and centrifuge at 4 °C for 10 min at 5000 rpm.
- Afterwards, completely remove the supernatant from the tube, add 3.5 mL of TE solution, and resuspend the protozoa pellet by vortexing or slow rounds of pipetting; then, divide it into aliquots of 1 mL and submit to DNA extraction, as reported below.
  **OPTIONAL STEP** We follow the proliferation of a cell population with regards to their DNA content using flow cytometry (BD Accuri C6 Plus).

### 3.2. DNA Extraction Methods

#### 3.2.1. Sodium Dodecyl Sulphate 20%/Triton X-100 (the Addition of Mixed Surfactant)

**Figure 3:**
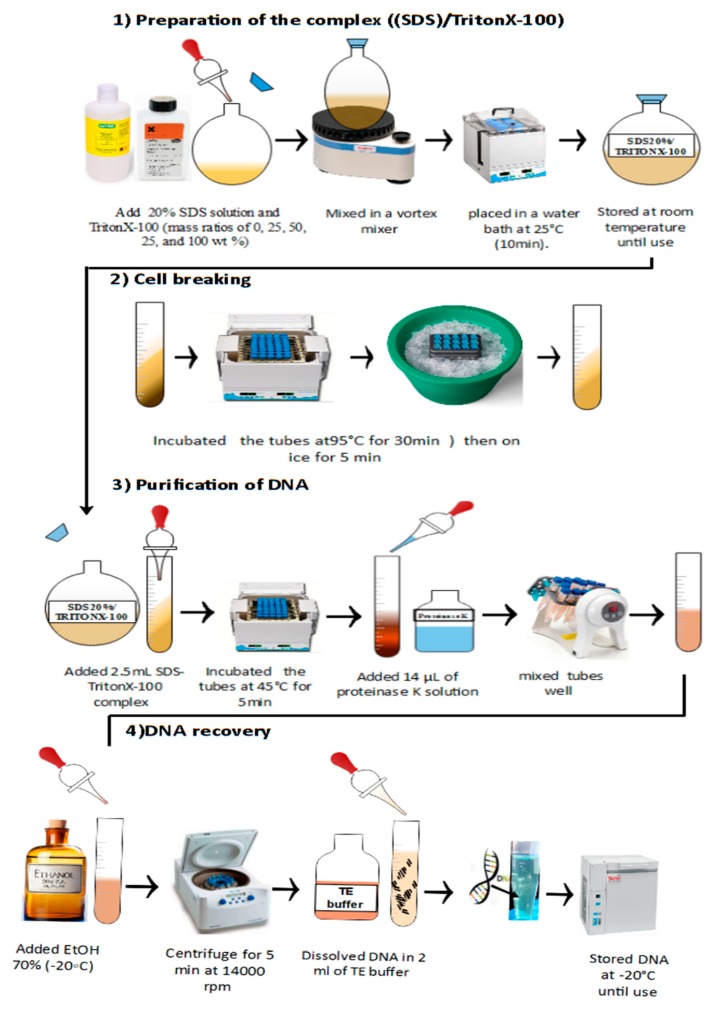
Schematic representation of the SDS 20%S/Triton X-100 method for the extraction and purification of DNA from *Tetrahymena pyriformis*.

1. Preparation of the 20% SDS solution/Triton X-100 complexes (time for fulfillment: 1 h, Figure 3)
  - To perform the SDS/Triton X-100 extraction method, the extraction complex 20% SDS solution/Triton X-100 was prepared beforehand.
    **OPTIONAL STEP** If sodium dodecyl sulfate (20% solution) is not available, it can be prepared with SDS solid. To prepare 100 mL of SDS (20% solution), weigh 20 g of SDS in a 250 mL conical flask/beaker. Add 80 mL deionised/Milli-Q water and mix, heat to 68 °C. Adjust the volume to 100 mL with deionised/Milli-Q water and mix again.
  - SDS (20% solution) and Triton X-100 (mass ratios of 0, 20, 40, 60, 80, and 100 wt %) were mixed intensively in a vortex mixer, and placed in a water bath at 25 °C for 45 min.
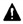
**CRITICAL STEP** If there SDS in the solvent precipitate, re-dissolve it by warming the mix at 60 °C for 10 min.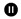 **PAUSE STEP** The mix can be stored at room temperature for several months. Do not store the SDS (20% solution) at 4 °C, because SDS will precipitate at temperatures below 15 °C.
2. Cell breaking using thermal shock methods (time for fulfillment: 30 min, Figure 3):
  - The tubes (1 mL protozoa pellet) were incubated in a water bath at 95 °C for 20 min (intermittently vortexing every 5 min).
  - The tubes were put on ice for 10 min.
3. Purification of DNA (time for fulfillment: 10 min, Figure 3):
  - A total of 2.5 mL of the SDS/Triton X-100/H_2_O complex was added to the tube.
  - The tubes were incubated in a water bath at 45 °C for 5 min.
  - After that, 14 µl of proteinase K solution (20 mg/mL) was added to the suspension, and the tubes were mixed well.
4. DNA recovery (time for fulfillment: 20 min, Figure 3)
  - First, 500 μL of 70% ethanol was added to the tube.
  - The tube was closed and inverted several times
  - The tubes were centrifuged at 14000 rpm (maximum speed) for 10 min at 25 °C.
  - The supernatant was completely removed.
    **OPTIONAL STEP** to remove the supernatant, one can decant the supernatant after the first centrifugation.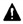 **CRITICAL STEP** Take care with this step, as the pellet sometimes does not adhere tightly to the tube and can be lost while removing the supernatant.
  - Liquid remains will be stuck on the wall of microcentrifuge tube.
  - A second flash spin is sufficient to collect all the liquid at the bottom, which can be then removed by pipetting.
  - The pellet was air dried for 5 min.
  - The traces of ethanol must then be removed, as they may inhibit some enzyme reactions and dissolve the pellet in 25 μL sterile double distilled water or TE (pH 8.0).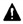 **CRITICAL STEP** Do not overdry the pellet; overdried pellets are difficult to dissolve.
    **OPTIONAL STEP** To dissolve the pellet, one can vortex the solution gently for a brief period and also incubate it at 37 °C for ∼20 min.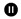 **PAUSE STEP** The solution can be stored at 4 °C for a few days or stored at −20 °C for years

#### 3.2.2. DNA Extraction by Chelex 100 Resin APD Complex and SDS–Chloroform

The extraction of DNA by the Chelex 100 matrix APD complex and SDS–chloroform methods was conducted as described in Table 2, and according to Fenicia et al. [8], Manaffar et al. [9], and Sambrook and Green [10].

### 3.3. Cost and Time Consumed

The cost for each method was estimated based on the price of chemicals, enzymes, and disposable items (including microfuge tubes and pipette tips) consumed for one extraction from a single pellet. The time required to finish one extraction from a single pellet for each method was estimated.

### 3.4. Evaluation of the Genomic DNA Quality, Quantity, and Integrity

A quantitative spectrophotometric assay of DNA was performed using NanoDrop and Qubit. Absorbance was measured at wavelengths of 260 and 280 (A_260_ and A_280_, respectively) nm. The absorbance quotient (A_260_/A_280_) provides an estimate of DNA purity. An absorbance quotient value of 1.8 < ratio (R) < 2.0 was considered to be good, purified DNA. A ratio of <1.8 is indicative of protein contamination, where as a ratio of >2.0 indicates RNA contamination. A total of 5 μL of DNA was run on a 1% agarose gel and visualized by UM illumination.

### 3.5. Pulsed-Field Gel Electrophoresis (PFGE)

Pulsed-field gel electrophoresis (PFGE) has been used extensively to prove that both compartments (micronucleus and macronucleus) have been isolated. PFGE was performed in a 1% agarose gel with 0.52 XTBE buffer (Tris-borate-EDTA) at 8 °C in a Rotaphor (Biometra). The voltage was set at 200 to 150 V/cm, and the pulse times ranged from 10 to 100 s (log). The pulse angle ranged from 120 to 110 (lin) with ramping. The electrophoresis lasted for 24 h. Afterward, the gel was stained with ethidium bromide in the same buffer [11].

### 3.6. Polymerase Chain Reaction Amplification of the *Metallothionein* (*MT*) Gene

PCR amplification of *metallothionein* (*MT*) gene was done in duplicates, using primers targeting the protist *Tetrahymena pyriformis (GL, L 1630/1)* (Table 1). Reaction mixtures contained 50 μL of 1× buffer KCl–MnCl2 (Fermentas), 1.5 mM MnCl2, 0.5 mM deoxynucleotide solution (dNTPs), 100 pM of each primer set, 1 μL Taq Polymerase Recombinant (1 U μL 174 −1) (Life Technologies, Nærum, Denmark), 350 ng of the extracted DNA, and 2.5 units of Taq DNA polymerase was prepared in a 300 μL PCR tube. The PCR conditions are described in Table 1. Subsequently, 5 μL of PCR products were analyzed by electrophoresis on 1% agarose gel in 1XTAE buffer.

### 3.7. Restriction Enzyme Digestion Analysis

The restriction digestion of the PCR products was carried out with the HindIll enzyme. The PCR products were exposed to digestion by the restriction endonuclease, in a total volume of 20 µL (10 µL PCR product, 2 µL enzyme buffers, 0.2 µL enzymes, and 7.8 µL distilled water), and placed in the incubator at 37 °C for 8 h. The digested products were analyzed on 2% agarose gel. The restriction products were analyzed by electrophoresis (Bio-Rad) on a 1% agarose gel, and the molecular weight of restricted fragments was analyzed by gel documentation systems (G-Box; Syngene) after ethidium bromide (Himedia) staining [12].

## 4. Expected Results

**Figure 4:**
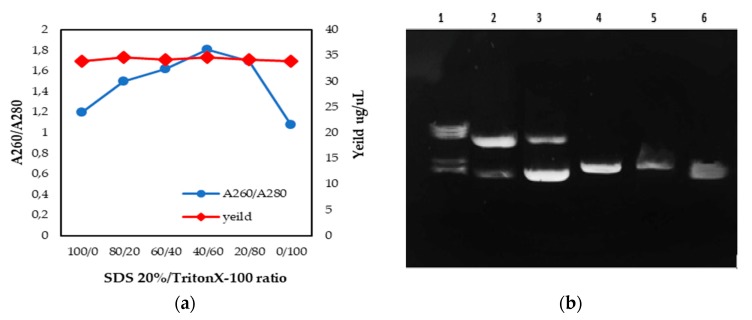
DNA was extracted and purified from *Tetrahymena pyriformis (GL, L 1630/1)* using the SDS 20%/TritonX-100 method. (**a**) Overview of DNA quality and quantity (NanoDrop), according to the SDS/Triton X-100 ratio—a description of what is contained in the second panel. (**b**) Electrophoretic analysis of *Tetrahymena pyriformis* DNA, isolated using various SDS/Triton X-100 ratios. Each lane indicates a different sample (Lane 1: 100/0; Lane 2: 80/20; Lane 3: 60/40; Lane 4: 40/60; Lane 5: 20/80; Lane 6: 0/100).

DNA was extracted from *Tetrahymena pyriformis (GL, L 1630/1)* using the supplementation of the mixed surfactant (SDS/Triton X-100) with different ratios (0%, 20%, 40%, 60%, 80%, 100%). The purity and yield of genomic DNA samples were estimated with NanoDrop for the purpose of evaluating parameters like quality and quantity (Figure 4a). The DNA yields were consistently approximately 34 µg/µL for all tested samples and replicates. In addition, the DNA was accepted as pure, since the A260/280 ratio was 1.8. Using this measurement method, the A260/280 ratio varied from 1.06–1.80. There was a linear correlation between the starting SDS/Triton X-100 ratio and the purity of the extracted DNA. Based on our results, we observed that the optimal SDS/Triton X-100 ratio for the successful extraction of DNA was 40% SDS/60% Triton X-100 (A260/A280 = 1.81).

The integrity of the extracted DNA was determined by gel electrophoresis. As shown in Figure 4b, lane 4 (40% SDS/60% Triton X-100) appeared to correspond to a high-quality extract. Lanes 1, 2, and 3 showed more than one band, and lanes 5 and 6 showed a non-pure band. This suggests that the DNA in lane 4 was neither contaminated with RNA nor degraded, compared to the DNA in the other lanes; this confirmed the results found with NanoDrop (A260/A280 = 1.81).

The efficiency of our SDS/Triton X-100 ratio was then compared with three widely-used alternative techniques, namely the Chelex 100 matrix APD complex and SDS–chloroform methods. As shown in Table 2, data analysis revealed that the time required (min), toxic compound, cost estimate per sample (in USD), and quality and quantity of the recovered DNA varied depending on the DNA extraction method. The highest quality of DNA (A260/A280) was obtained by the SDS/Triton X-100 and Chelex 100 matrix methods (1.81 and 1.84, respectively, where a ratio of approximately 1.8 is accepted as pure DNA). In addition, the maximum yield was obtained with the SDS/Triton X-100 method (34.75 and 33.77 ug/ul for NanoDrop and Qubit, respectively), while no DNA was detected for the APD complex method. The required time for different methods uses for DNA extraction varied from 2 h up to 6.4 h. The SDS/Triton X-100 method was the fastest compared to other methods used, although no toxic compound was use and the cost estimate per sample ˂ $3 USD.

**Figure 5:**
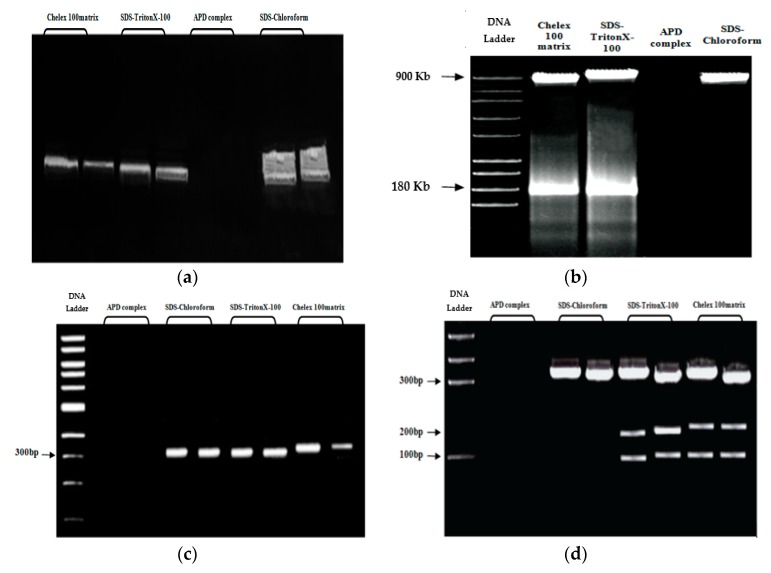
Electrophoretic analysis (**a**), pulsed-field gel electrophoresis (PFGE) analysis (**b**), electrophoresis analysis of the PCR product (**c**), and electrophoresis analysis of the polymerase chain reaction (PCR) product after digestion with HindIll restriction enzyme (**d**) of DNA extracted from *Tetrahymena pyriformis* using various extraction methods.

Figure 5a shows the DNA results visualized by agarose gel electrophoresis. The extracts from the Chelex 100 matrix (lanes 1 and 2) and SDS/Triton X-100 (lanes 3 and 4) methods generated clean single bands, while the SDS-Chloroform method (lanes 7 and 8) gave a larger range of fragment sizes. Regarding the APD complex method, no positive bands were detected in the gel electrophoresis (lanes 5 and 6).

To prove that both compartments (micronucleus and macronucleus) have been isolated, the DNA extracts from all methods were further subjected to pulsed-field gel electrophoresis (PFGE). As shown in Figure 5b, PFGE generated two bands—one for macronuclear DNA, with a size of 180 kb, and very prominent band for micronuclear DNA, with a size of 900 kB. In a previous study, the macronuclear DNA of *Tetrahymena* was found to fluctuate between 100kb to 1500 kb, while the micronuclear DNA is much larger [13,14,15].

In order to confirm their suitability for amplification, DNA extracts were subsequently subjected to PCR analysis for *metallothionein* (*MT*) gene-specific primers. Figure 5c shows the results of PCR amplification of DNA isolated from *Tetrahymena pyriformis* (*GL, L 1630/1*) using different extraction methods. All extracts had positive amplification of the *metallothionein* gene (310 bp), except for the DNA extracted by the APD complex method (Lanes 1–2).

The restriction digestion of the PCR products was carried out. As it is seen in Figure 5d, digestion of a 310 bp fragment of *metallothionein* (*MT*) gene by HindIll restriction endonuclease generated tree clear bands (310, 220, 104 bp) for the Chelex 100 matrix and SDS/Triton X-100 methods and one band (310 bp) for the SDS–Chloroform method, while no band was observed for the APD complex method.

## 5. Conclusions

Here, we report a simple method for the extraction and purification of DNA from *Tetrahymena pyriformis*. The efficiency of this technique was compared to three widely-used alternative techniques, namely the Chelex 100 matrix APD complex and SDS–chloroform methods. We demonstrated that the improved method using SDS/Triton X-100 was the easiest and most cost-effective, and was more suitable for molecular applications, such as the extraction and purification of DNA from *Tetrahymena pyriformis* compared to the other methods.

## Figures and Tables

**Table 1:** Primer sequences, final concentrations and annealing temperatures for the primers used for measuring the degradation of DNA.

Primer Set (Reference)	Sequence (5′–3′), (3′–5′)	Denaturing (°C; min)	Annealing (°C; min)	Elongation (°C; min)	N° cycles
Metallothionein (MT) primers [12]	CGTGAATAAAATGGATAAGGTTAATAA, CATTTGCAACATTCACAAGTCTTAC	94; 3.5	55; 0.4	72;0.3	30

**Table 2:** Summary of results from comparative study of DNA extraction methods.

DNA Extraction Methods	DNA Extraction Protocol Stage				Time Required (min)	Toxic Compound	Cost Per Sample (US$)	A260/280 1	NanoDrop µg/µL	Qubit µg/µL
	Cell Lysis	Denaturation of Nucleoproteins	Removal of Contaminants	DNA Precipitation						
SDS-TritonX100	Thermal shock	Proteinase K	SDS/Triton X-100	Ethanol	2 h	None	˂ 3	1.81	34.75	33.77
Chelex100matrix	Heat	Chelex/Proteinase K	Chelex	Isopropanol	3 h	Isopropanol	˂ 3	1.84	2.62	2.08
APD complex	SDS	Proteinase K	Ammonium Pyrrolidine Dithiocarbamate	Ethanol	3 h	None	˃ 3	ND	ND	ND
SDS-chloroform	SDS	Phenol/Proteinase K	Phenol-chlorofom isoamyl alcohol	Ethanol	6 h 40 min	Phenol, chloroform	˂ 3	1.08	30.87	28.79

ND: not detected. ^1^ A ratio of approximately 1.8 is accepted as pure DNA. Lower values might be related to the presence of phenol, or other reagent residues used in the DNA extraction. ^2^ SDS/Triton X-100: this SDS and Triton X-100 ratio (40/60) provided the best result.

## References

[1] Darcy P., Kelly J.P., Leonard B.E., Henry A.B. (2002). The effect of lofepramine and other related agents on the motility of *Tetrahymena pyriformis*. Toxicol. Lett..

[2] Leick V., Bog-Hansen T.C., Christensen S.T., Kaufman S.J. (1996). Concanavalin A Receptors and the Chemosensory Behaviour of Tetrahymena thermophila. Exp. Biol. Online.

[3] Yu S., Geng J., Zhou P., Wang J., Chen X., Hu J. (2008). New hydroxyapatite monolithic column for DNA extraction and its application in the purification of Bacillus subtilis crude lysate. J. Chromatogr..

[4] Kan C.W., Fredlake C.P., Doherty E.A.S., Barron A.E. (2004). DNA sequencing and genotyping in miniaturized electrophoresis systems. Electrophoresis.

[5] Barbosa C., Nogueira S., Gadanho M., Chaves S. (2016). DNA extraction: Finding the most suitable method. Molecular Microbial Diagnostic Methods.

[6] Fenicia L., Anniballi F., De Medici D., Delibato E., Aureli P. (2007). SYBR Green real-time PCR method to detect Clostridium botulinum type A. Appl. Environ. Microbiol..

[7] Pousada R.C., Cyrne M.L., Hayes D. (1979). Characterization of Preribosomal Ribonucleoprotein Particles from Tetrahymena pyriformis. Eur. J. Biochem..

[8] Walsh P.S., Metzger D.A., Higuchi R. (2013). Chelex 100 as a Medium for Simple Extraction of DNA for PCR-Based Typing from Forensic Material. BioTechniques.

[9] Manaffar R., Maleki R., Zare S., Agh N., Soltanian S., Sehatnia B., Sorgeloos P., Bossier P., Van G. (2010). A New Method for Rapid DNA Extraction from Artemia (Branchiopoda, Crustacea). Int. J. Bioeng. Life Sci..

[10] Green M.R., Sambrook J. (2012). Molecular cloning. A Laboratory Manual.

[11] Carle G.F., Frank F., Olson M.V. (1986). Electrophoretic separations of large DNA molecule by periodic inversion of electric field. Science.

[12] Cheung A., Pok L., Vincent K.L.L., King M.C. (2005). Tilapia metallothionein genes: PCR-cloning and gene expression studies. Biochim. Biophys. Acta.

[13] Conover R.K., Brunk C.F. (1986). Macronuclear DNA Molecules of Tetrahymena thermophila. Mol. Cell. Biol..

[14] Yao M.C., Gorovsky M.A. (1974). Comparison of the DNA sequences of Tetrahymena macro- and micronuclei. Chromosoma.

[15] Yao M.C., Yao C.H. (1981). Repeated hexanucleotide C-C-C-C-A-A is present near free ends of macronuclear DNA of Tetrahymena. Proc. Natl. Acad. Sci. USA.

